# Application of 3D-Printed Orthopedic Cast for the Treatment of Forearm Fractures: Finite Element Analysis and Comparative Clinical Assessment

**DOI:** 10.1155/2020/9569530

**Published:** 2020-07-25

**Authors:** Yanjun Chen, Hui Lin, Qinqin Yu, Xiaodong Zhang, Defeng Wang, Lin Shi, Wenhua Huang, Shizhen Zhong

**Affiliations:** ^1^Guangdong Engineering Research Center for Translation of Medical 3D Printing Application, Guangdong Provincial Key Laboratory of Medical Biomechanics, School of Basic Medical Sciences, Southern Medical University, Guangzhou, China; ^2^Guangdong Innovation Platform for Translation of 3D Printing Application, The Third Affiliated Hospital of Southern Medical University, Southern Medical University, Guangzhou, China; ^3^School of Mechanical and Electrical Engineering, Putian University, Fujian, China; ^4^Research Institute for Frontier Science, Beihang University, Beijing, China; ^5^Beijing Advanced Innovation Center for Big Data-Based Precision Medicine, Beijing, China; ^6^Hangzhou Innovation Institute of Beihang University, Hangzhou, China; ^7^Department of Imaging and Interventional Radiology, The Chinese University of Hong Kong, Shatin, Hong Kong

## Abstract

**Objective:**

This pilot study is aimed at investigating the mechanical characteristics of a cast-wrapped fractured forearm and performing a clinical comparative study of our own developed 3D-printed orthopedic cast.

**Methods:**

An integrated finite element (FE) model including a forearm and a 3D-printed cast wrapping the forearm was created. The distal radial ulna in this model was cut through to mimic the bone fracture. A 400 N force and 1 Nm rotation moment, which were much larger than the loading conditions encountered in daily life for a human being, were applied on the palm. We conducted a comparative clinical study by using statistical assessment. 60 patients with forearm fractures were selected and treated with manual reduction and external fixation cast. All patients were divided into three groups with equal members (20): (a) 3D-printed external cast group, (b) traditional plaster external fixation group, and (c) splint external fixation group. The clinical efficacy, wrist function, and patient satisfaction were scored and compared.

**Results:**

In the condition of 400 N loading, the fracture displacements in anterior-posterior (AP), posterior-anterior (PA), medial to lateral (ML), and lateral to medial (LM) compression directions were 1.2648, 1.3253, 0.8503, and 0.8957 (mm), respectively, and the corresponding fracture stresses were 4.5986, 3.9129, and 5.0334, 7.9197 (MPa), respectively. In the inward (IR) and outward (OR) rotations, the fracture displacements were both 0.02628 (mm), and the corresponding fracture surface stresses were 0.1733 and 0.1723 (MPa), respectively. In the clinical efficacy, wrist function, and patient comfort evaluation, the total scores of group A were both higher than those in groups B and C (*P* < 0.05).

**Conclusion:**

A 3D-printed orthopedic cast was capable of exerting appropriate mechanical correction loads on specific areas to maintain optimal alignment of a fractured forearm and thus could achieve the favorable clinical efficacy and patient comfort.

## 1. Introduction

The 3D printing technology in the orthopedic field is growing swiftly on account of its advantages in personalizing features and rapid manufacture [1–5]. For nonsurgically treatment of fractures, the concept of using 3D printing technology to make a personalized cast with an appropriate fit and a ventilated structure for patients has also emerged [6]. To gain a 3D-printed cast, technicians can use the image data with 3D spatial information of limbs captured by the 3D scanner or medical imaging devices and then conduct the computer-aided design to obtain the Stereolithography (STL) format file for 3D printing. For the pressure more appropriately distributing [7], a 3D-printed cast is expected to be more comfortable during the treatment. In addition to the benefit from personalized design and use of lightweight materials for 3D printing, the novel cast is more fashionable and portable as well. Although 3D printing had made advances in the development of casting techniques, most published works for application of 3D-printed casts were still in the concept stage or initial phase [8–10], speaking to the need for experimental data and clinical experiences.

Forearm fractures are common skeletal injuries and occur at all ages especially in children and in the elderly, and distal radius fractures are the most common type [11, 12]. Cast immobilization is preferred in the majority of patients with nonsurgically treated forearm fractures [13]. Poor ventilation and improper fit present in traditional casts are associated with treatment complications. Our previously published studies developed a novel cast fabricated by 3D printing [14] and gained preliminary clinical experiences, which was the first clinical experience reported.

Some previous studies showed that the finite element (FE) analysis in medicine helped surgeons better understand global biomechanical features of injured tissues and involving medical devices [15–17]. The overall biomechanical traits of a cast-wrapped injured forearm during the treatment period are still unclear. For the computer-designed cast, FE simulation could predict changes in stress distribution and fracture displacement in the overall range of movement. As far as we know, rare studies were published to globally depict the biomechanical features of the fractured bone of forearm with an orthopedic cast.

The objective of this pilot study is firstly to develop an integrated FE model of a cast-wrapped injured forearm to provide an engineering insight of treatment efficacy and secondly to further perform a comparative clinical study including our own developed cast and common conventional cast, plaster cast, and splint.

## 2. Materials and Methods

### 2.1. Finite Element Modeling

A three-dimensional FE model was developed to simulate the external cast fixation in the forearm fracture model and to simulate the different loading tests.

#### 2.1.1. Model of the Forearm and Cast

**Figure 1:**
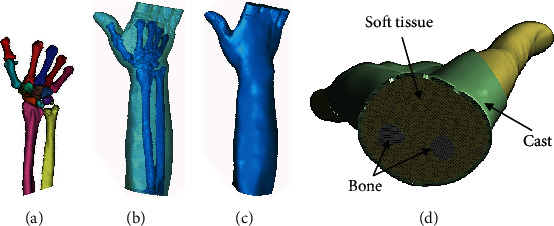
The main process of modeling: (a) image segmentation; (b, c) reverse-engineering reconstruction; (d) final assembly model.

**Figure 2:**
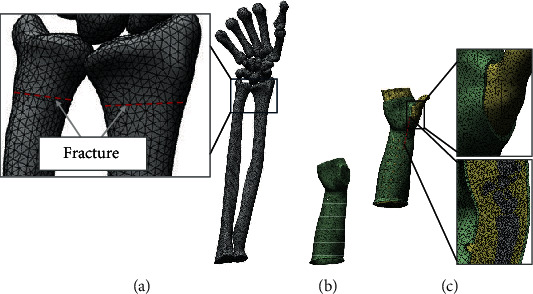
The hole forearm and cast FE model: (a) model of bones, in which distal radioulnar fracture was set; (b) model of cast; (c) final assembly mesh model, with a magnification and a section box.

Forearm modeling was done using computed tomography (CT) volume data of a volunteer, and the 3D model was then created from the segmented portion of the CT scan slices using Mimics10.01 (Materialise, Belgium). The 3D models of the ulna, radius, carpal, and metacarpal bones were obtained, and the soft tissues were modeled using Boolean operations to build the forearm model. The cast model with a holed surface pattern for the consideration of ventilation was generated using the system developed by our previously published study [6, 14] and assembled with the forearm model using Solidworks 2015 (Dassault, France), and we finally get the complete geometric model. Hence, a forearm composed cast model could be used for the model to enable the simulation of cast immobilization for the forearm fracture.  Figure 1 shows the main process of modeling. The final model was then imported to Workbench 18.0 (ANSYS, USA) for FEA, in which tetrahedral element solid187 was used to divide the mesh, and the unit sizes were 1 mm for bones, 3 mm for the soft tissue, and 1 mm for the cast (Figure 2). The number of assembly units was 419901, and the number of nodes was 645171. The distal radioulnar fracture was set as transverse fractures at the level about 3 cm from the distal articular surface using the surface cutting function of the software (Figure 2(a)).

#### 2.1.2. Material Property

In this study, the bone, soft tissue, and cast model were set as homogeneous linear elastic materials as shown in Table 1 [18–20]. Binding contact was assumed between the cast and soft tissue and between soft tissue and bone, and frictionless contact was assumed between fracture surfaces.

#### 2.1.3. Mechanical Loading Set

**Figure 3:**
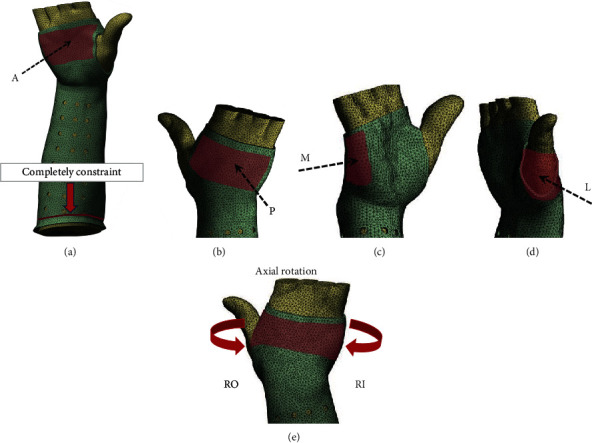
The boundary condition of the FE model. (a–d) The compression force perpendicular to the palm (400 N) along the AP, PA, ML, and LM directions, respectively; (e) rotation moment with 1 Nm toward RO and RI, respectively.

This study mainly simulated the effect of the cast in immobilizing the fractures and did not consider the status of longitudinal compression. The region nearby the proximal side of the cast was fixed. For the setting of axial load, some previous studies used 100 N load or 1 Nm bending moment [21, 22], but in this study, a larger load was applied to evaluate the immobilization performance of the cast. A compression load with 400 N was exerted on the palm along anterior to posterior (AP), posterior to anterior (PA), medial to lateral (ML), and lateral to medial (LM) directions to mimic different mechanical scenarios. A 1 Nm [23] rotation moment toward the inward (RI) and outward (RO) of the palm was applied to the top end side of the cast to test the biomechanical influence of shear forces (Figure 3). The mechanical force and rotation moment were much larger than the loading conditions encountered in daily life for a human being. The stress distribution and fracture displacement under different working conditions were observed.

### 2.2. Clinical Application Evaluation

The institutional review board of our hospital approved this prospective study (ID: 201603006). Written informed consent was obtained for the study inclusion of each subject.

#### 2.2.1. Study Population

Between August 2016 and May 2018, a total of 60 patients who suffered forearm fractures were enrolled in this study (24 males, 36 females). All cases were treated with external casting from the middle and upper forearm to the palm, with an age ranging from 5 to 78 years, including Colles' fracture (46 cases), Smith's fracture (12 cases), and ulnoradial diaphyseal fracture (2 cases).  Table 2 shows the distribution of different types of fractures in each group. The inclusion criteria for this study were as follows: (1) forearm stable fractures that can receive conservative treatment and (2) following the doctor's instructions for regular visits. The exclusion criteria were as follows: (1) with preexisting bone disease (such as tumor, metastases, or metabolic disorder), (2) previous history of fracture of the same limb, and (3) with any skin break or compound fracture.

#### 2.2.2. Grouping

The subjects were divided into three groups according to the casting methods: group A using 3D-printed cast, group B using plaster cast, and group C using splint fixation, with 20 subjects in each group. The grouping criteria are as follows: (1) 60 numbers were randomly divided into three groups (20 numbers in each group); (2) the patients were numbered according to the chronological order of the first visit, and the numbered patients were assigned to the corresponding groups; and (3) the informed consent of each enrolled patient was fully obtained. All patients first underwent closed reduction, groups A and B used traditional plaster cast fixation, and group C used splint fixation. 3D-printed orthopedic casts were applied to patients in group A after one week for 3D printing design and manufacturing.

#### 2.2.3. 3D Printing

To obtain workable data for later cast design, both forearms of patients in group A were scanned by a CT imaging system (Aquillion 64, Toshiba, Japan) or MR (Achieva 1.5 or 3.0 T, Philips, Netherlands) imaging equipment. All the patients lied on the scanning bed of the scanning system and then raised their hands above their heads with palms facing up. Both hands with symmetric postures were scanned to obtain raw data. Data from the other forearm without injury became the alternative due to the plaster casting of the injured forearm. Patients' raw models were inputted into our cast design system to perform patient-specific design as per clinical requirements. Polyamide (PA2200) was employed in the 3D printing fabrication of all casts using a selective laser sintering (SLS) 3D printer EOS P395 (Germany).

#### 2.2.4. Postprocessing for 3D-Printed Cast

**Figure 4:**
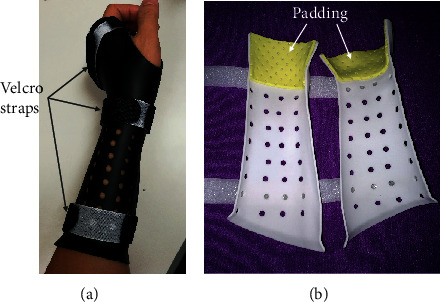
3D-printed cast assembled to a patient's forearm. (a) Velcro straps are used as fixing devices; (b) padding on the specific anatomical regions close to the wrist.

It included padding the medial surface, mechanical grinding or rolling sharp edges, and adding fixation components. Velcro straps were adopted as fixation straps by mounting on the cast to adjust the assembly and create a cast that is tailor-fitted to an injured limb. Cushion pads were glued on the distal regions of the cast medial surface to avoid local high pressure and scratching of the skin (Figure 4).

#### 2.2.5. Clinical Trial Assessment

Two questionnaires were designed including two groups of survey questions concerning the clinical efficacy (Table 3) and patient satisfaction (Table 4) for all patients based on the clinical test and published studies [24, 25]. The first questionnaire was completed by the surgeon who examined patients after six weeks of follow-up. With the assistance from doctors (only explaining the details of the questionnaire to let the patient fully understand questions without any personal recommendations to affect selection), patients completed the second assessment questionnaire after six weeks of casting. The Cooney modification of the Green and O'Brien score [26] was applied for the wrist functional assessment after 3 months, including the evaluation of pain, functional status, range of motion, and grip strength (the appendix).

### 2.3. Statistical Analysis

Numerical data were reported as the mean ± standard deviation (SD) except for the FEA calculation results. Differences between overall groups were compared using one-way ANOVA followed with the Bonferroni post hoc multiple comparisons, the LSD method was used for multiple comparisons with equal variances, and the Dunnett T3 method was used for multiple comparisons with heterogeneous variances. *P* values of <0.05 were regarded as significant. All analyses (except Bland-Altman plots) were performed using SPSS 23.0 (IBM SPSS, Armonk, NY).

## 3. Results

### 3.1. Finite Element Analysis Results

#### 3.1.1. Displacement

Under the constraint condition of proximal forearm and cast completely fixed, global displacements of the distal end were more obvious than the displacements of the bone. The maximum global displacement was 12.685 mm in the AP direction loading condition, but the maximum sliding displacement of the fracture surface was only 1.325 mm in the PA direction. The global and fracture surface displacements were the least under the rotating condition. In general, the stress of the skeleton was greater than that of the cast, and the stress of the fracture surface was less than that of the skeleton and cast.  Table 5 shows a summary of finite element analysis results.

#### 3.1.2. Stress

**Figure 5:**
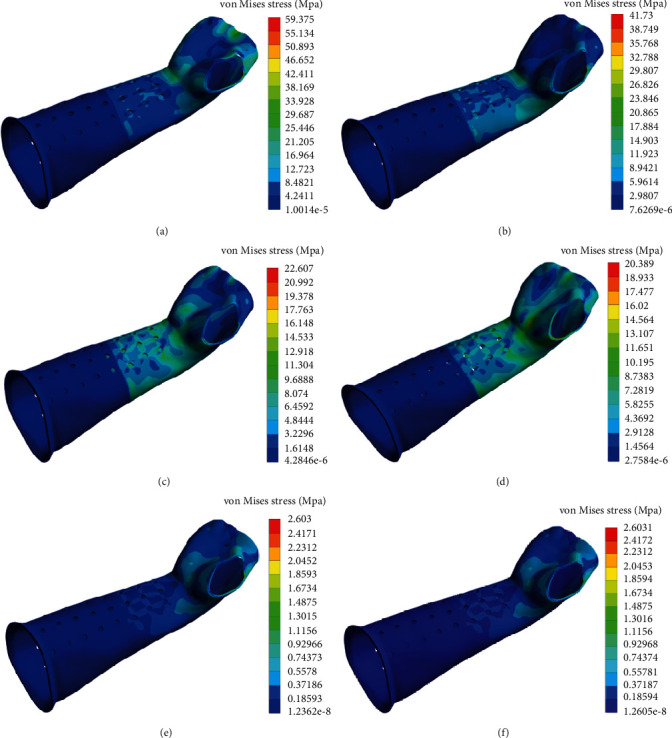
Stress (von Mises) contour of the cast under different loading conditions. (a–d) The stress distribution of the cast under the load of 400 N in the AP, PA, ML, and LM loading directions; (e, f) the stress distribution of the cast in IR and OR direction under 1 Nm rotation moment.

**Figure 6:**
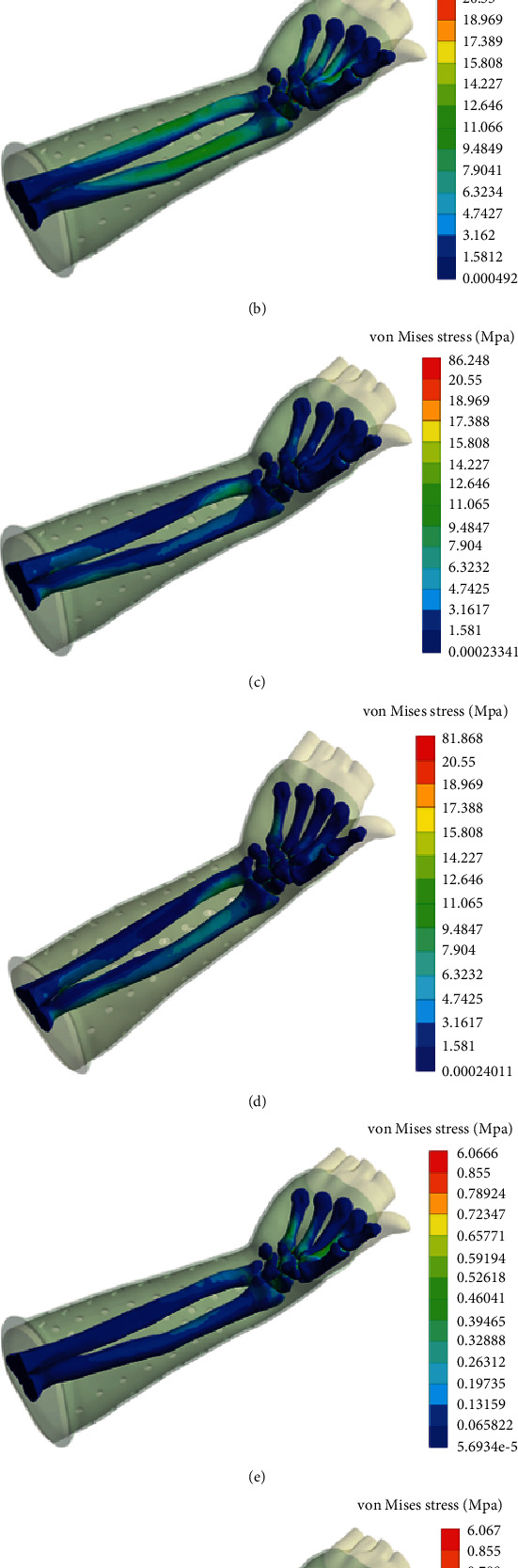
Stress (von Mises) contour of the bones under different loading conditions. (a–d) The stress distribution of the bones under the load of 400 N in the AP, PA, ML, and LM loading directions; (e, f) the stress distribution of the bones in IR and OR direction under 1 Nm rotation moment.

As the proximal forearm and cast were set completely fixed, the loading site was located at the palm. The stress distribution for the cast was mainly in the middle and the distal section under different loading conditions (Figure 5). For the bone, stress was mainly distributed in the ulnar and radial diaphysis and metacarpal bones (Figure 6). No overconcentrated stress distribution could be found in all loading conditions.

### 3.2. Clinical Evaluation

#### 3.2.1. Basic Outcome of the Patients

60 cases (age ranging from 5 to 78 years; 24 males and 36 females) of distal forearm fractures were treated with external casting. All the patients achieved the purpose of clinical treatment. The novel cast as well as plaster and splinting maintained fracture bone alignment and immobilizing the forearm during the healing process. No patient underwent secondary reduction. Moreover, no breakage occurred in any cast during the treatment period.

#### 3.2.2. Assessment of Clinical Effectiveness

The questionnaire assessment of clinical efficacy was completed by the surgeon for each patient in our study. The total score was 10.20 ± 0.951 for group A, 9.10 ± 1.119 for group B, and 9.35 ± 1.137 for group C. Although the scores of each item did not show a significant difference between the groups (for all, *P* > 0.05), the total score differences of the three groups were statistically significant (*P* = 0.005) (Table 6). For the wrist functional assessment, the Green and O'Brien score (Cooney modification) was applied and the fineness rate was 80% for group A, 65% for group B, and 70% for group C (Table 7).

#### 3.2.3. Assessment of Patient Satisfaction

The questionnaire assessment of patient satisfaction was completed, and the result showed that the total score for group A was 8.65 ± 1.040, 6.85 ± 1.137 for group B, and 8.10 ± 1.252 for group C, and the difference was statistically significant (*P* ≤ 0.001). Group A scored higher than the other two groups for comfort, and group C scored higher than the other two groups for odor and skin itchiness (*P* < 0.05, for all) (Table 8).

## 4. Discussion

Plaster casting and splinting are widely used for nonsurgical treatment of forearm fracture after reduction, but complications including cutaneous diseases, compartment syndrome, and vascular comprise have been reported due to unbalanced pressures and high stiffness [27, 28]. We sought to determine if a novel cast fabricated by 3D printing technology based on the patient limb feature could be applied to immobilize the bone after the closed reduction and minimize the risks of complications. We found that the 3D-printed cast developed from a patient's images could hold the fracture reduction at a proper anatomic position and spread the pressure evenly, suggesting a custom-fit immobilization during the entire treatment process.

Although the forces were more complicated in actual motions, the loss of reduction during the immobilizing period was mainly caused by shear forces [29]. Therefore, we mimicked 6 different loading conditions including AP, PA, ML, LM, IR, and OR in the FEA to calculate the displacement and stress. Furthermore, mechanical loads applied in each condition were much higher than the actual daily scenario. The simulation results demonstrated that the maximum global displacement was 12.685 mm in AP direction and it was distal to the limb; the maximum relative displacement of the fracture surface was only 1.325 mm in the PA direction, suggesting that the effect of immobilization was significant even under high loading condition. For the pressure distribution, the results showed that the stresses in AP, PA, ML, and LM loading conditions were much higher than rotating conditions, ranging from 20.389 to 59.375 MPa for the cast and from 81.868 to 174.720 MPa for the bones. No stress concentration was observed from the von Mises stress contour. The results of this study confirmed that the custom-fit structure of the novel cast was capable of reducing the risk of high local pressures and improving comfort.

To the best of our knowledge, this is the first comparative study on the application of 3D-printed cast and traditional cast. All patients participating in each group had completed the entire therapeutic course without negative clinical consequences. The total score of the clinical efficacy assessment and the fineness rate of the functional evaluation showed superior outcomes in group A compared to the other two groups. Wearing pressures were necessary for any casting or splinting technologies to maintain the reduction of the fracture and perform orthopedic corrections effectively [7]. The 3D-printed casts we designed enlarged the contact area between the cast and skin and thus applied appropriate orthopedic pressure on the injured limb. Compared with that in group A, the pink skin was more common in groups B and C often found in those regions with high local pressure and motion-related scratch. Proper and early functional exercise might have rehabilitation benefits to restore the function of the wrist for patients with distal forearm fractures [13, 30]. Given that the Velcro straps were adopted as fixation straps, it was convenient to adjust, disassemble, and assemble the 3D-printed cast. The fixation structure created a favorable condition for patients to carry out wrist functional exercises. For patient satisfaction evaluation, patient compliance scored lower in group A than the other two groups. It was mainly because the novel cast had never been used in clinical practice, and thus, most patients were wary and had an acclimation time. The moderate contact like a fitted sleeve covering, evenly distributed pressure, made the score of patient comfort in group A higher than in groups B and C significantly, as well as the total score. The ventilated structure was able to keep the space dry between the skin and the cast. Therefore, the scores of cast odor and skin itchiness in groups A and group C were excellent, while group B had a lower score compared with the other two.

The strengths of our study included the use of FEA to simulate the casting effectiveness under different loading conditions, objective evaluation of clinical efficacy, and follow-up on fracture outcomes, as well as the reasonable patient satisfaction assessment. Although not on the basis of clinical cases with fractures at different anatomical sites, the results were likely to be representative of routine clinical practice and the fracture type typically referred for nonsurgical treatment. One drawback of the 3D-printed cast was poor timeliness. It took about 3-5 days for a cast to be designed and manufactured (including shipping time), so the patient had to use a plaster cast or splinting temporarily during this period. It was also worth noting that patient compliance or treatment adherence was a challenge for physicians to carry out this research due to the skepticism of the technology since it had not been employed in clinical applications before. Importantly, all the patients in group A appreciated the advantages after one or two weeks of application of the novel cast.

There are some limitations in this study. First, in the FE model, bone and soft tissues were simplified as the linear isotropic material. The computational accuracy in specific regions with large deformities would be relatively low due to the nonlinear properties of soft tissues under large displacement. Nevertheless, the FEA provided global insight into the biomechanical profile to gain optimal management of forearm fracture [31, 32]. Second, as a single-center study, the small sample size might result in statistical bias for clinical evaluation. Studies with a large sample size would be designed to clarify the efficacy of the novel cast in subjects with forearm fracture as well as fractures in other parts of the limb. Third, the relatively high fabrication cost of a 3D printing cast restricted its application. As a pilot study, we were mainly concerned with the clinical feasibility of the application of the 3D-printed cast compared with a traditional plaster cast and splint.

This study demonstrated that the patient-specific 3D-printed cast with a wearer-friendly design not only played an important role to maintain the alignment of forearm fractures but also improved patient comfort and reduced risk of complications. Cast design and 3D-printing fabrication required a relatively long time, so the proposed method could not be used in emergency situations currently. The rapid growth of 3D printing technologies would reduce the fabrication cost in the near future. Further assessment of the practical use of this technology would be integrated into clinical workflows for prospective validation in the consideration of acceptance by physicians and patients.

## 5. Conclusions

The biomechanical analysis concluded the potential better treatment experiences by using our own developed 3D-printed cast based on the following engineering pieces of evidence: spreading the high concentrated stress to induce pressure-related complications and maintaining an optimal alignment for favorable bone healing by applying appropriated correction forces. The further clinical trials confirmed the favorable efficacy and treatment experience by using the novel cast and thus supported the biomechanical conclusions. Furthermore, the comparative clinical study demonstrated the clinical advantages of casting technology over the traditional counterparts on patient adherence and satisfaction.

## Figures and Tables

**Table 1:** Material attribute assignment.

Material	Elastic modulus (MPa)	Poisson's ratio	Element type
Bone	13400	0.3	Tetrahedron element
Soft tissue	0.15	0.45	Tetrahedron element
Cast	1500	0.3	Tetrahedron element

**Table 2:** Distribution of different types of fracture.

Fracture type	3D-printed cast (group A)	Plaster cast (group B)	Splinting (group C)	Total
Colles	16 (26.7)	15 (25.0)	15 (25.0)	46 (76.7)
Smith	3 (5.0)	5 (83.3)	4 (6.7)	12 (20.0)
Ulnoradial diaphyseal	1 (1.7)	0 (0)	1 (1.7)	2 (3.3)

Note: data are presented as the cases (%).

**Table 3:** Assessment of clinical effectiveness.

Assessment item	Assessment contents and grading standard			
	Excellent—3	Good—2	Acceptable—1	Poor—0
Stability of immobilization	No loss of reduction	Slight shift but no need for remanipulation	Reenforced the same cast	Loss of reduction requiring further procedure
Blood circulation	Good terminal circulation with a florid complexion	Venous obstruction relief after physical movement or arm lifting	Pale skin, low temperature of the arm	Significant ischemia of involved limb, compartment syndrome
Wear pressure-related pain	No pain	Slight pain with a minor influence of sleep	Mild pain causes poor-quality sleep	Severe pain causes difficulty falling asleep
Pressure sores	No abnormality of the skin	Nonblanchable erythema of the intact skin	Skin breakdown or bleeding blister	Full-thickness skin loss

**Table 4:** Assessment of patient satisfaction.

Item	Assessment contents and grading standard			
	Excellent—3	Good—2	Acceptable—1	Poor—0
Patient comfort	Very comfortable	Occasional irritation	Not comfortable but endure	Bad experience of wearing the cast
Patient compliance	Strong willing	Minor doubt	Dubious but complied	Accepting reluctantly
Cast odor and smell	None	Slight cast odor	Smelly cast after heavy sweating	Stinky cast
Skin itchiness	No itch	Rarely itchy	Frequent itch but tolerable	Severely itchy

**Table 5:** Finite element analysis results.

Loading condition	Displacement (mm)				Stress (MPa)		
	Global	Cast	Bone	Fracture surface	Cast	Bone	Fracture surface
AP	12.685	11.959	6.824	1.265	59.375	174.720	4.599
PA	6.655	6.168	5.887	1.325	41.730	146.780	3.913
ML	3.437	3.123	2.706	0.850	22.607	86.248	5.033
LM	3.536	3.427	2.686	0.896	20.389	81.868	7.920
IR	0.226	0.226	0.166	0.026	2.603	6.067	0.173
OR	0.226	0.226	0.166	0.026	2.603	6.067	0.172

Note: data were generated by software calculation (no standard deviation).

**Table 6:** Clinical efficacy evaluation results.

Group	Scoring item				Total
	Stability of immobilization	Blood circulation	Wear-pressure-related pain	Pressure cores	
A	2.75 ± 0.444	2.50 ± 0.513	2.30 ± 0.470	2.65 ± 0.587	10.20 ± 0.951
B	2.75 ± 0.444	2.05 ± 0.510	1.95 ± 0.394	2.35 ± 0.489	9.10 ± 1.119
C	2.80 ± 0.410	2.10 ± 0.447	2.15 ± 0.587	2.30 ± 0.470	9.35 ± 1.137
*F*	0.089	4.698	2.566	2.670	5.783
*P*	0.915	0.015	0.086	0.078	0.005

**Table 7:** Green and O'Brien score (Cooney modification) evaluation results.

Group	*n*	Excellent	Good	Fair	Poor	Fineness rate
A	20	12 (60.0)	5 (25.0)	3 (15.0)	0	85.0
B	20	7 (35.0)	6 (30.0)	5 (25.0)	2 (10.0)	65.0
C	20	7 (35.0)	7 (35.0)	4 (20.0)	1 (5.0)	70.0

Note: data are presented as cases (%). Fineness rate = (excellent + good)/*n*, presented as %; *P*^AB^ = 0.014, *P*^AC^ = 0.035, and *P*^BC^ = 0.329.

**Table 8:** Patient satisfaction evaluation results.

Group	Scoring item				Total
	Comfort	Compliance	Odor	Itchiness	
A	2.70 ± 0.470	1.85 ± 0.875	1.90 ± 0.718	2.20 ± 0.523	8.65 ± 1.040
B	1.45 ± 0.605	2.10 ± 0.553	1.50 ± 0.513	1.80 ± 0.410	6.85 ± 1.137
C	1.76 ± 0.639	1.95 ± 0.510	2.05 ± 0.510	2.35 ± 0.587	8.10 ± 1.252
*F*	26.685	0.700	4.666	6.164	12.950
*P*	≤0.001	0.503	0.013	0.004	≤0.001

## Data Availability

The data used to support the findings of this study are included in the article.

## Appendix

### A.1. Green and O'Brien Score (Cooney Modification)

(I) Pain (25 points)
  - 25: none
  - 20: mild and occasional
  - 15: moderate and tolerable
  - 0: severe or intolerable
(II) Range of motion (25 points): flexion+extension and percentage of normal
  - 25: 100
  - 15: 75-99
  - 10: 50-74
  - 5: 25-49
  - 0: 0-24
(III) Grip strength (25 points): percentage of normal
  - 25: 100
  - 15: 75-99
  - 10: 50-74
  - 5: 25-49
  - 0: 0-24
(IV) Activities (25 points)
  - 25: returned to regular employment
  - 20: restricted employment
  - 15: able to work but unemployed
  - 0: unable to work because of pain
(V) Final result
  - 90-100: excellent
  - 80-89: good
  - 65-79: fair
  - <65: poor

## References

[1] Ganguli A., Pagan-Diaz G. J., Grant L. (2018). 3D printing for preoperative planning and surgical training: a review. *Biomedical Microdevices*.

[2] López-Torres I. I., Sanz-Ruíz P., León-Román V. E., Navarro-García F., Priego-Sánchez R., Vaquero-Martín J. (2019). 3D printing in experimental orthopaedic surgery: do it yourself. *European Journal of Orthopaedic Surgery & Traumatology*.

[3] Ranjan N., Singh R., Ahuja I. P. S. (2020). On 3D printed scaffolds for orthopedic tissue engineering applications. *SN Applied Sciences*.

[4] Golovin M. A., Marusin N. V., Golubeva Y. B. (2018). Use of 3D printing in the orthopedic prosthetics industry. *Biomedical Engineering*.

[5] Punyaratabandhu T., Liacouras P. C., Pairojboriboon S. (2018). Using 3D models in orthopedic oncology: presenting personalized advantages in surgical planning and intraoperative outcomes. *3D Printing in Medicine*.

[6] Lin H., Shi L., Wang D. (2016). A rapid and intelligent designing technique for patient-specific and 3D-printed orthopedic cast. *3D Printing in Medicine*.

[7] Rizza R., Liu X., Thometz J., Tassone C. J. (2015). Comparison of biomechanical behavior between a cast material torso jacket and a polyethylene based jacket. *Scoliosis*.

[8] Fitzpatrick A. P. (2017). Design of a patient specific, 3D printed arm cast. *KnE Engineering*.

[9] Kuppu Rao G., Shah T., Dayanand Shetty V., Ravi B. (2017). Custom design & fabrication of 3D printed cast for ankle immobilisation. *KnE Engineering*.

[10] Graham J., Wang M., Frizzell K., Watkins C., Beredjiklian P., Rivlin M. (2020). Conventional vs 3-dimensional printed cast wear comfort. *Hand*.

[11] Mauck B. M., Swigler C. W. (2018). Evidence-based review of distal radius fractures. *The Orthopedic Clinics of North America*.

[12] MacIntyre N. J., Dewan N. (2016). Epidemiology of distal radius fractures and factors predicting risk and prognosis. *Journal of Hand Therapy*.

[13] Delft E. A. K. V., Gelder T. G. V., Vries R. D., Vermeulen J., Bloemers F. W. (2019). Duration of cast immobilization in distal radial fractures: a systematic review. *Journal of Wrist Surgery*.

[14] Chen Y., Lin H., Zhang X., Huang W., Shi L., Wang D. (2017). Application of 3D–printed and patient-specific cast for the treatment of distal radius fractures: initial experience. *3D Printing in Medicine*.

[15] Lekadir K., Noble C., Hazrati-Marangalou J. (2016). Patient-specific biomechanical modeling of bone strength using statistically-derived fabric tensors. *Annals of Biomedical Engineering*.

[16] Liu W., Yang L., Kong X. (2017). Stiffness of the locking compression plate as an external fixator for treating distal tibial fractures: a biomechanics study. *BMC Musculoskeletal Disorders*.

[17] Stricker A., Widmer D., Gueorguiev B., Wahl D., Varga P., Duttenhoefer F. (2018). Finite element analysis and biomechanical testing to analyze fracture displacement of alveolar ridge splitting. *BioMed Research International*.

[18] Cheung J. T., Zhang M., An K. N. (2004). Effects of plantar fascia stiffness on the biomechanical responses of the ankle-foot complex. *Clinical Biomechanics*.

[19] Jasiuk I., Abueidda D. W., Kozuch C., Pang S., Su F. Y., McKittrick J. (2018). An overview on additive manufacturing of polymers. *JOM*.

[20] He Y., He J., Wang F. (2015). Application of additional medial plate in treatment of proximal humeral fractures with unstable medial column: a finite element study and clinical practice. *Medicine*.

[21] Caiti G., Dobbe J. G. G., Bervoets E. (2019). Biomechanical considerations in the design of patient-specific fixation plates for the distal radius. *Medical & Biological Engineering & Computing*.

[22] Rogge R. D., Adams B. D., Goel V. K. (2002). An analysis of bone stresses and fixation stability using a finite element model of simulated distal radius fractures. *The Journal of Hand Surgery*.

[23] Nassiri M., Macdonald B., O'Byrne J. M. (2013). Computational modelling of long bone fractures fixed with locking plates - how can the risk of implant failure be reduced?. *Journal of Orthopaedics*.

[24] Grafstein E., Stenstrom R., Christenson J. (2010). A prospective randomized controlled trial comparing circumferential casting and splinting in displaced Colles fractures. *CJEM*.

[25] Inglis M., McClelland B., Sutherland L. M., Cundy P. J. (2013). Synthetic versus plaster of Paris casts in the treatment of fractures of the forearm in children: a randomised trial of clinical outcomes and patient satisfaction. *Bone & Joint Journal*.

[26] Kwok I. H., Leung F., Yuen G. (2011). Assessing results after distal radius fracture treatment: a comparison of objective and subjective tools. *Geriatric Orthopaedic Surgery & Rehabilitation*.

[27] Delasobera B. E., Place R., Howell J., Davis J. E. (2011). Serious infectious complications related to extremity cast/splint placement in children. *The Journal of Emergency Medicine*.

[28] Davis D. I., Baratz M. (2010). Soft tissue complications of distal radius fractures. *Hand Clinics*.

[29] Ketata H., Affes F., Kharrat M., Dammak M. (2019). A comparative study of tapped and untapped pilot holes for bicortical orthopedic screws – 3D finite element analysis with an experimental test. *Biomedical Engineering / Biomedizinische Technik*.

[30] van Delft E., Bloemers F. W., Sosef N. L., Bonjer H. J., Schep N., Vermeulen J. (2019). Dislocated distal radial fractures in adult patients: 4 weeks versus 6 weeks of cast immobilisation following reduction, a multicentre randomised controlled trial, study protocol. *BMJ Open*.

[31] Caruso G., Tonon F., Gildone A. (2019). Below-elbow or above-elbow cast for conservative treatment of extra-articular distal radius fractures with dorsal displacement: a prospective randomized trial. *Journal of Orthopaedic Surgery and Research*.

[32] Gamba C., Fernandez F., Llavall M. C., Diez X. L., Perez F. S. (2017). Which immobilization is better for distal radius fracture? A prospective randomized trial. *International Orthopaedics*.

